# Impact of DPP4 Inhibitors in Survival of Patients With Prostate, Pancreas, and Breast Cancer

**DOI:** 10.3389/fonc.2020.00405

**Published:** 2020-03-31

**Authors:** Chintan Shah, Young-Rock Hong, Rohit Bishnoi, Azka Ali, William Paul Skelton, Long H. Dang, Jinhai Huo, Nam H. Dang

**Affiliations:** ^1^Division of Hematology and Oncology, Department of Medicine, University of Florida, Gainesville, FL, United States; ^2^Department of Health Services Research, Management and Policy, College of Public Health and Health Professions, University of Florida, Gainesville, FL, United States; ^3^Division of Medical Oncology, H. Lee Moffitt Cancer Center, University of South Florida, Tampa, FL, United States; ^4^Ochsner Medical Center, Baton Rouge, LA, United States

**Keywords:** DPP4 (Dipeptidyl peptidase 4) inhibitors, cancer, sitagliptin, Januvia, prostate, breast, pancreas

## Abstract

**Background:** Dipeptidyl peptidase-4 (DPP4), a cell surface protein, exhibits a crucial role in tumor biology and regulation of the immune system. We aim to study the impact of DPP4 inhibitors (DPP4i) in patients with prostate cancer (PRC), pancreatic cancer (PC) and breast cancer (BC).

**Methods:** Using the SEER and Medicare linked database, we identified patients with PRC or PC or BC with coexisting type II diabetes mellitus between 2007 and 2015. Patients were classified into four groups: (1) not on either DPP4i or metformin (reference group), this group included patient that were on anti-diabetic agents other than metformin or DPP4i (2) metformin only, (3) DPP4i only, and (4) DPP4i along with metformin (combination group). Overall survival (OS) analyses were performed using SAS®, version 9.4.

**Results:** We identified 15,330 patients with PRC, 5,359 patients with PC and 16,085 patients with BC. In PRC cohort, patients on DPP4i had significant survival advantage with HR 0.77 (95% CI: 0.64–0.93), *P* = 0.005 when compared to the reference group. Patients taking metformin also had significant OS benefit with HR 0.87 (95% CI: 0.81–0.93), *P* < 0.0001 when compared to the reference group. However, in BC cohort, OS did not favor the patients taking DPP4i with HR 1.07 (95% CI: 0.93–1.25, *P* = 0.33). Similarly, in PC cohort, OS was indifferent for the patients on DPP4i with HR 1.07 (95% CI: 0.93–1.24, *P* = 0.68). Upon subgroup analyses of PRC patients, the survival favored the group taking DPP4i, irrespective of stage, use of chemotherapy, androgen-deprivation therapy, and prostatectomy or radiation therapy.

**Conclusions:** DPP4i seems to improve survival in PRC patients; however, not in PC or BC patients. While the exact mechanism involved remains to be elucidated, a prospective clinical trial would help to confirm these findings.

## Introduction

Cluster of differentiation 26 (CD26) is a multifunctional type II transmembrane serine peptidase which is present at low density on resting T lymphocytes and is up-regulated upon T lymphocyte activation. CD26 has an extracellular domain with DPP4 (dipeptidyl peptidase IV) enzymatic activity and a short cytoplasmic domain (1). A truncated form (sCD26/DPP4) is also present in serum and other body fluids (2). CD26/DPP4 exerts its immune-mediated and non-immune-mediated activities via various mechanisms such as its role in T lymphocyte activation and as a costimulatory interacting protein, which results in enhanced T cell effector functions; its role as a proteolytic enzyme and signal transduction mediator; as well as its role in adhesion and cell motility. Moreover, CD26/DPP4 appears to have a role in tumor biology, with its expression levels being associated with cancer progression and tumor malignant behavior (1–5).

Inhibition of DPP4 also prevents inactivation of glucagon like peptide-1 which in turn leads to the secretion of insulin and better glycemic control. As a result of this mechanism of action, DPP4 inhibitors (DPP4i) are approved and are used in diabetes mellitus type 2 as monotherapy as well as in combination with metformin. Several previous studies have also examined the relationship between new cancer initiation and the use of DPP4i, but no consistent relationships have been found. A large meta-analysis of 72 trials and a randomized controlled trial which specifically examined new cancer as a primary outcome did not show any significant association between use of DPP4i and cancer initiation (6, 7).

The role of CD26/DPP4 in prostate cancer is not yet well-understood. *In-vitro* studies showed that the blockage of CD26 in 1-LN tumor cell lines led to a decrease in tumor cell invasiveness (8). Another study using prostate cancer xenograft model showed that the DPP4 gene was down-regulated during the progression to castration-resistant prostate cancer, suggesting its tumor suppressive property (9). However, no studies have evaluated the clinical outcome of using DPP4i in prostate cancer patients. Similarly, the role of CD26/DPP4 in breast cancer remains poorly understood. *In-vitro* studies demonstrated thatinhibition of CD26/DPP4 stimulated breast cancer metastasis, likely via induction of CXCL12/CXCR4 (10), while others reported inhibition of CD26/DPP4 led to the suppression of breast cancer tumor growth (11). To evaluate the role of CD26/DPP4 inhibition in clinical setting, we conducted a retrospective analysis of patients with advanced airway and colorectal cancers with diabetes who were taking DPP4i (12). The study showed significant advantage in progression-free survival and a positive trend in overall survival (OS); however, OS did not reach the level of statistical significance likely due to small sample size (12). To further clarify the role of DPP4i, we conducted a SEER (Surveillance Epidemiology and Endpoint Research)-Medicare analysis of colorectal cancer and lung cancer patients, which also showed a similar trend toward beneficial effects associated with CD26/DPP4 inhibition (13). Apart from colorectal and lung cancer, CD26/DPP4 protein is well-expressed in prostate cancer cells, while its expression in pancreatic or breast cancer cells is relatively lower (1, 2, 14). In this present work, we aim to assess the impact of CD26/DPP4 inhibition in patients with prostate, pancreatic and breast cancerthrough the use of a national database.

## Methods

We utilized the SEER-Medicare database for our study. SEER database represents ~34% of the U.S. population and is maintained by the National Cancer Institute (www.seer.cancer.gov) of the National Institutes of Health (15). The Medicare database is maintained by the Centers for Medicare and Medicaid Services for eligible US residents, and it comprise of over 97% of the US population aged 65 years or older. The database provides individual patient level demographic and survival data from the SEER cancer registry in conjunction with comprehensive therapeutic information from the Medicare program (16).

### Cohort Selection

**Figure 1:**
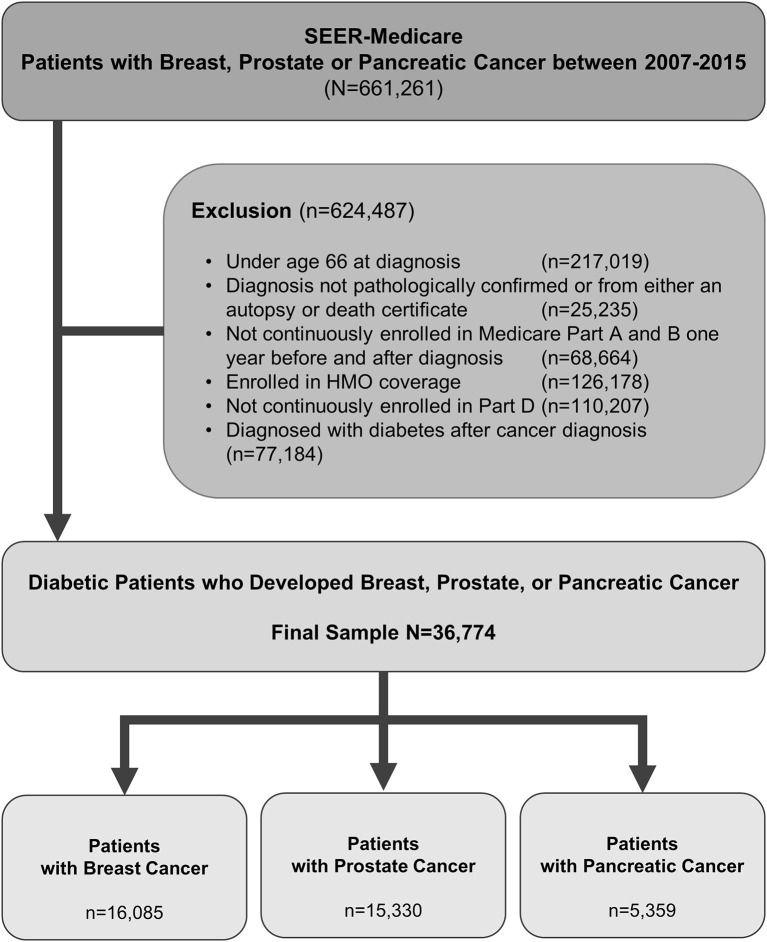
Criteria used and flow chart of patient selection with prostate, pancreas, or breast cancer.

By using International Classification of Diseases for Oncology, third edition (ICD-O-3) codes, we identify patients who were diagnosed with prostate cancer, or pancreatic cancer, or breast cancer and diabetes mellitus type 2 between 2007 and 2015. Patients were older than 65 years as the data source is SEER-Medicare. The study samples were restricted to those with continuous Medicare Part A and Part B insurance coverage and no HMO coverage 12 months before and 12 months after a cancer diagnosis or until death. Figure 1 shows the flowchart of patient selection with the detailed criteria used. By using generic name and National Drug Codes in SEER-Medicare Part D file, we identified use of DPP4i in our patient cohort. DPP4i such as, alogliptin, linagliptin, saxagliptin, sitagliptin, and vildagliptin were selected. Similarly, use of metformin was identified. Table 1 shows characteristics of included patients. We used ICD (ninth revision) procedure codes, level II Healthcare Common Procedure Coding System (HCPCS), and Current Procedural Terminology (CPT) codes in the Medicare claims to identify treatment rendered within 1 year of cancer diagnosis. We used the modified algorithm proposed by Klabunde et al. to calculate the Charlson Comorbidity Index (17, 18).

**Table 1:** Baseline Characteristics of Study Cohort by DDP4 inhibition.

		**DDP4**				
**Characteristic**	**Total**	**No**	**%**	**Yes**	**%**	***p*-value**
**Demographics**						
Year of diagnosis						<0.0001
2007	2,161	2,127	6.3	34	1.1	
2008	3,713	3,544	10.6	169	5.2	
2009	3,891	3,700	11.0	191	5.9	
2010	3,844	3,592	10.7	252	7.8	
2011	4,147	3,795	11.3	352	10.9	
2012	4,245	3,771	11.2	474	14.7	
2013	4,737	4,179	12.5	558	17.3	
2014	4,935	4,326	12.9	609	18.9	
2015	5,101	4,520	13.5	581	18.0	
Age group						0.0873
65–69	9,246	8,445	25.2	801	24.9	
70–74	10,848	9,861	29.4	987	30.7	
75–79	8,146	7,409	22.1	737	22.9	
80+	8,534	7,839	23.4	695	21.6	
Sex						0.9957
Male	17,692	16,143	48.1	1,549	48.1	
Female	19,082	17,411	51.9	1,671	51.9	
Race/ethnicity						<0.0001
Non-hispanic white	24,795	22,733	67.8	2,062	64.0	
Non-hispanic black	4,937	4,564	13.6	373	11.6	
Hispanic	2,757	2,412	7.2	345	10.7	
Others	4,285	3,845	11.5	440	13.7	
Marital status						0.1466
Single	6,855	6,279	18.7	576	17.9	
Married	17,389	15,814	47.1	1,575	48.9	
Other	12,530	11,461	34.2	1,069	33.2	
Census poverty						0.3801
0– <5% poverty	7,393	6,712	20.0	681	21.1	
5– <10%	9,038	8,253	24.6	785	24.4	
10–20%	10,721	9,789	29.2	932	28.9	
20–100%	9,203	8,424	25.1	779	24.2	
Unknown	419	376	1.1	43	1.3	
Census region						<0.0001
West	14,407	13,098	39.1	1,309	40.7	
Northeast	8,472	7,588	22.6	884	27.5	
Midwest	4,653	4,402	13.1	251	7.8	
South	9,224	8,450	25.2	774	24.1	
Rural/urban status						<0.0001
Urban area	32,155	29,253	87.2	2,902	90.1	
Rural area	4,619	4,301	12.8	318	9.9	
Charlson comorbidity index						<0.0001
0	5,075	4,999	14.9	76	2.4	
1	14,636	13,351	39.8	1,285	39.9	
2	7,765	6,978	20.8	787	24.4	
3+	9,298	8,226	24.5	1072	33.3	
Cancer type						<0.0001
Breast	16,085	14,777	44.0	1,308	40.6	
Prostate	15,330	14,096	42.0	1,234	38.3	
Pancreas	5,359	4,681	14.0	678	21.1	
Stage						<0.0001
I	6,781	6,188	18.4	593	18.4	
II	17,365	15,957	47.6	1,408	43.7	
III	2,123	1,918	5.7	205	6.4	
IV	5,019	4,463	13.3	556	17.3	
Unknown	5,486	5,028	15.0	458	14.2	
Surgery						<0.0001
No	19,502	17,701	52.8	1,801	55.9	
Yes	17,272	15,853	47.2	1,419	44.1	
Chemotherapy						0.0008
No	24,575	22,508	67.1	2,067	64.2	
Yes	12,199	11,046	32.9	1,153	35.8	
Radiotherapy						0.0015
No	22,654	20,587	61.4	2,067	64.2	
Yes	14,120	12,967	38.6	1,153	35.8	
Insulin						<0.0001
No	29,978	27,552	82.1	2,426	75.3	
Yes	6,796	6,002	17.9	794	24.7	
Sulfonylurea						<0.0001
No	27,032	25,291	75.4	1,741	54.1	
Yes	9,742	8,263	24.6	1,479	45.9	

*DPP4, dipeptidyl peptidase*.

### Statistical Analysis

Metformin is commonly used for the management of DM-II. To evaluate the impact of DPP4i or metformin independently and in combination, patients were classified into four groups based on the use of DPP4i and metformin: (1) not on either agent (reference group), this group included patients that were on anti-diabetic agents other than metformin or DPP4i (2) metformin only, (3) DPP4i only, and (4) DPP4i along with metformin (combination group). Cox Proportional Hazards survival model was used to assess the overall survival (OS) of these groups, controlling for patients demographic and clinical characteristics. In subgroup analysis, we compared patients on DPP4i only against the reference group. Bivariate analyses compared baseline characteristics between patients taking DPP4i and not, using Pearson chi-square tests. The OS time was defined as from the date of cancer diagnosis until the date of death or loss of follow-up. Statistical significance was defined as a *P* < 0.05. All analyses were performed using SAS software (version 9.4; SAS Institute, Cary, NC, USA). The University of Florida institutional review board approval was obtained.

## Results

**Figure 2:**
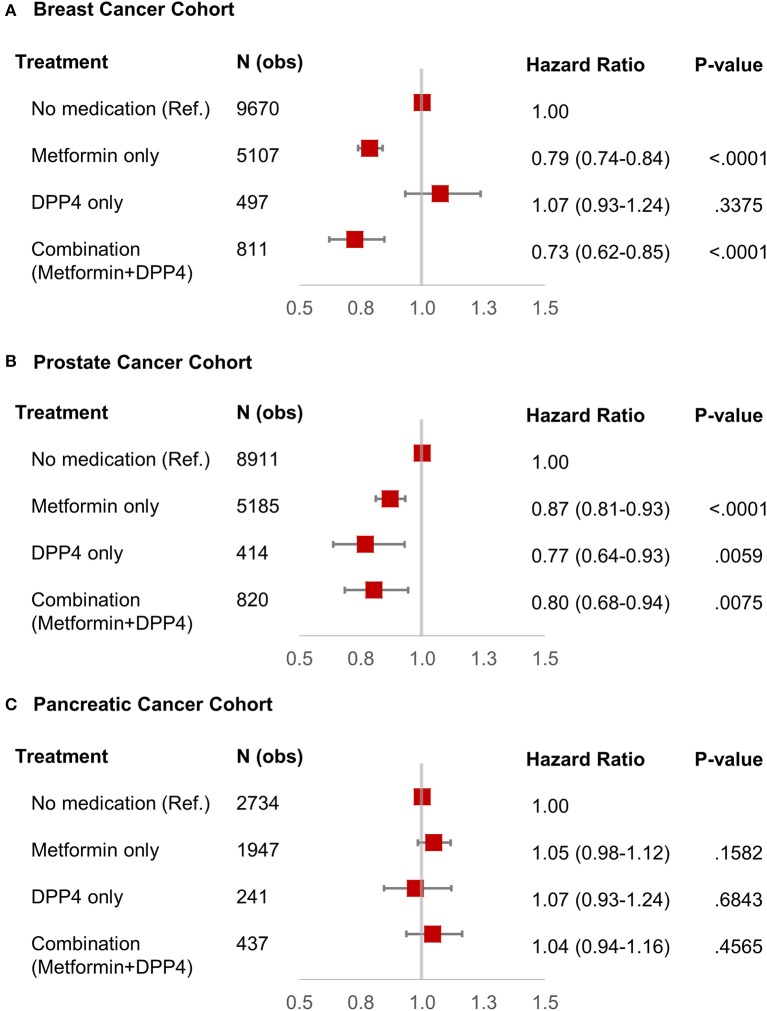
Forest plot with hazard ratio showing survival analysis of breast cancer **(A)**, prostate cancer **(B)**, and pancreatic cancer **(C)** patients.

We identified 16,085 breast cancer patients that met our inclusion criteria. Table 1 shows the characteristics of selected patients. A total of 9,670 (60.11%) patients were not on metformin or DPP4i (reference group), while 5,107 (31.75%) patients were on metformin, 497 (3.08%) patients were on DPP4i, and 811 (5.04%) patients were on the metformin and DPP4i combination. As shown in Figure 2A, the patients treated with metformin showed significant OS benefit with HR 0.79 (95% CI: 0.74–0.84), *P* < 0.001 when compared to the reference group. Similarly, patients on metformin and DPP4i combination also showed a significant survival benefit with HR 0.73 (95% CI: 0.62–0.85), *P* < 0.001 when compared to the reference group. However, survival did not favor the patients who were only on DPP4i with HR 1.07 (95% CI: 0.93–1.25, *P* = 0.33).

**Figure 3:**
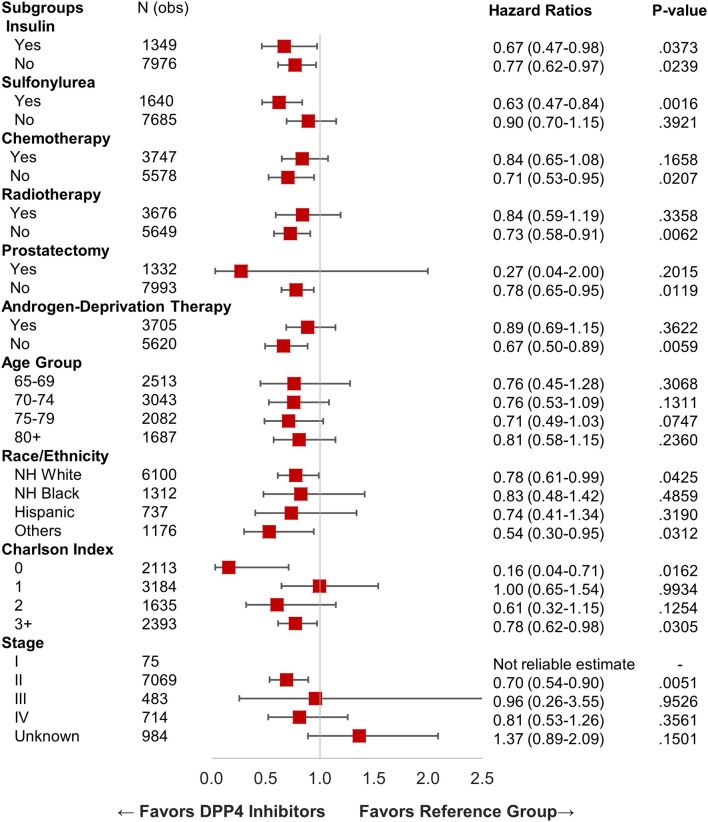
Forest plot with hazard ratio showing survival analysis of various subgroups within prostate cancer patients.

We identified 15,330 patients with prostate cancer who met our inclusion criteria. Table 1 shows the characteristics of selected patients. A total of 8,911 (58.12%) patients were not on metformin or DPP4i (reference group), while 5,185 (33.82%) patients were on metformin, 414 (2.7%) patients were on DPP4i, and 820 (5.34%) patients were on the metformin and DPP4i combination. The group who was on metformin showed significant OS benefit with HR 0.87 (95% CI: 0.81–0.93), *P* < 0.0001 when compared to the reference group. Similarly, patients on DPP4i also showed a significant survival benefit with HR 0.77 (95% CI: 0.64–0.93), *P* = 0.005 when compared to the reference group. Patients who were on a combination of metformin and DPP4i also showed survival advantage compared to the reference group HR 0.80 (95% CI: 0.68–0.94), *P* = 0.007 (Figure 2B). For subgroup analyses, we only compared patients on DPP4i against reference group, to avoid confounding by metformin. It demonstrated the trend toward a beneficial effect of DPP4i, irrespective of stage (stage I, NR; stage II, HR 0.81; stage III, NR; stage IV, 0.76), treatments with chemotherapy (HR 0.83 with chemotherapy and HR 0.70 without chemotherapy), androgen-deprivation therapy (ADT) (HR 0.87 with ADT and HR 0.71 without ADT), prostatectomy (HR 0.50 with prostatectomy and HR 0.77 with no prostatectomy), or radiation (HR 0.89 with radiation therapyand HR 0.64 without radiationtherapy). However, statistical significance was not reached for the majority of them likely due to low sample size (Figure 3).

We identified 5,359 patients with pancreatic cancer who met our inclusion criteria. Table 1 shows the characteristics of selected patients. A total of 2,734 (51%) patients were not on metformin or DPP4i (reference group), while 1,947 (36.33%) patients were on metformin, 241 (4.5%) patients were on DPP4i, and 437 (8.15%) patients were on metformin and DPP4i combination. As shown in Figure 2C, none of these groups showed any beneficial effects on OS when compared to the reference group. Subgroup analysis was not performed as DPP4i did not show any beneficial effect in this group of patients.

## Discussion

To the best of our knowledge, this is the first review of a large national database looking into the impact of DPP4i on the survival of prostate, pancreatic and breast cancer patients.

By using the SEER-Medicare database, we showed that the prostate cancer patients who were on DPP4i had better survival compared to those who were not treated with DPP4i. The survival advantage shown in patients with prostate cancer taking DPP4 inhibitors only (HR 0.77; 95% CI: 0.64–0.93; *P* = 0.005) shows the benefit independent of metformin. However, the survival advantage of DPP4i was not evident in pancreatic cancer and breast cancer patients. We believe that this pattern may likely be at least partly due to the expression profile of CD26/DPP4. Protein Atlas of immunohistochemistry data by antibody staining of various normal as well as cancerous human cells showed that several prostate cancers and a few renal cell carcinomas displayed moderate to strong membranous or cytoplasmic positivity to antibody to CD26/DPP4, while most other cancers including pancreatic and breast did not (14). Moreover, CD26/DPP4 biochemical activity was found to be twice as high in prostate cancer compared to benign prostate hyperplasia tissues (19), which could be a responsible factor for the growth of prostate cancer cells. Furthermore, in an analysis of prostate cancer tissue samples from 494 patients, high expression of CD26/DPP4 was associated with poor prognosis, *P* < 0.001 (20). Taken all together, the blockage of DPP4 could have resulted in improved survival in prostate cancer patients in our analysis.

There are several potential mechanisms proposed for the role of DPP4i in cancer cells. Immunological function of CD26/DPP4 includes activation of resting T cells, costimulatory effects on T cells and signal transduction leading to increased secretion of cytotoxic granzymes such as TNF-α, IFN-γ, FAS-ligand (1). Our analyses imply that the anti-tumor activities of DPP4i in solid tumors are unlikely to be solely due to immunologic modulation, as these effects of DPP4i should not be dependent on the expression levels of DPP4 on the organ tissue. Moreover, CD26/DPP4 has a known role in metastasis. CD26 acts as a receptor for plasminogen 2ϵ, which stimulates matrix metalloproteinase-9 (MMP), leading to the degradation of the extracellular matrix required by cells to invade (8, 21). Blocking the DPP4 activity may thus lead to delayed propagation of cancer cells. We also performed various subgroup analyses in patients with prostate cancer (as shown in the results section). While we found an encouraging trend toward the survival benefit favoring the DPP4i cohort, the statistical significance was not reached, likely secondary to small sample size. A study using a larger sample size or prospective trials might help replicate our findings.

Given its role in cancer biology and the results of multiple preclinical studies, the first in human phase I clinical trial was conducted using a humanized antibody to CD26 (YS110) in malignant mesothelioma patients (22) and reported prolonged disease stabilization with good drug tolerance. One of the side effects with commercially available DPP4i is hypoglycemia, even though less commonly seen compared to sulfonylurea. Interestingly, hypoglycemia was not one of the commonly reported adverse effects in this phase I trial using YS110. Serum DPP4 level can be determined by assays measuring enzymatic cleavage of known DPP4 substrate. The level of inhibition of DPP4 by >80% that was found in this trial with YS110 was comparable to the oral administration of commercially available DPP4i.

The role of metformin as an anti-tumor agent is well-established in many types of cancer including prostate cancer (23–25). Metformin has antineoplastic effects such as adenosine monophosphate-activated protein kinase (AMPK)-dependent suppression of androgen signaling pathway, and alterations of insulin-like growth factor-1 (IGF-1) signaling pathways that cause the growth and proliferation of prostate cancer. Moreover, metformin increases the number of CD8^+^ tumor-infiltrating lymphocytes and also protects them from apoptosis and exhaustion which is characterized by decreased production of IL-2, TNFα, and IFNγ (26). Our analysis showed the OS benefit of metformin (HR 0.87) in prostate cancer patients and OS benefit was also evident in metformin and DPP4i combination group (HR 0.80). Further studies should explore if there is any synergistic activity of these two drugs in treatment of prostate cancer. The role of metformin in breast cancer patients is controversial as per published reports so far (27). Our analysis did show improved OS in breast cancer patients taking metformin (HR 0.81, *P* < 0.0001) but not in pancreatic cancer patients.

Our analysis has several limitations; mainly that it is a retrospective study which carries its own inherent biases. The sample size was another limitation for subgroup analyses. DPP-4 inhibitors are typically used as a second or third line in those who do not achieve adequate glycemic control to sulfonylurea, metformin, or a thiazolidinedione (28) leading relatively small sample size. SEER database includes data from 19 different geographical areas covering ~34% of the US population. Therefore, both data and results could be affected by regional trends in diagnosis and treatment of various disease as well as access to health care in those particular geographical areas. And thus caution should be exercised before generalization.

In conclusion, use of CD26/DPP4 inhibitors is associated with improved survival outcomes in patients with prostate cancer but not in breast or pancreatic cancer patients, which may be linked to the protein expression profiling of CD26/DPP4 in these malignancies. A well-designed prospective trial would assist in confirming these findings.

## Data Availability Statement

The datasets analysed for this study can be found in the SEER-Medicare database maintained by the National Cancer Institute (www.seer.cancer.gov).

## Ethics Statement

The studies involving human participants were reviewed and approved by University of Florida. Written informed consent for participation was not required for this study in accordance with the national legislation and the institutional requirements.

## Author's Note

The study presented here is the expansion of our previous work using the SEER-Medicare database which was submitted to the American Society of Clinical Oncology (ASCO) conference, June 2019 (29). The data and results presented here are different from our previous work as sample size and study duration differs. Moreover, the present study also includes patients with breast cancer.

## Author Contributions

CS and Y-RH had an equal contribution, participated in the study design, writing, and editing manuscript. ND and LD conceptualize the idea. Y-RH and JH provided data and performed data analysis. RB, AA, and WS participated in study design, writing, and editing manuscript. All authors helped with editing and finalizing the manuscript.

### Conflict of Interest

The authors declare that the research was conducted in the absence of any commercial or financial relationships that could be construed as a potential conflict of interest.
